# Sampling and quenching of CHO suspension cells for the analysis of intracellular metabolites

**DOI:** 10.1186/1753-6561-7-S6-P42

**Published:** 2013-12-04

**Authors:** Judith Wahrheit, Elmar Heinzle

**Affiliations:** 1 Biochemical Engineering Institute, Saarland University, D-66123 Saarbrücken, Germany

## Background

Metabolic studies are of fundamental importance in metabolic engineering approaches to understand cell physiology and to pinpoint metabolic targets for process optimization. Knowledge on intracellular metabolites, in particular in combination with powerful dynamic metabolic flux analysis methods will substantially expand our basic understanding on metabolism, e.g. about metabolic compartmentation [1]. Few protocols for quantitative analysis of intracellular metabolites in mammalian suspension cells have been proposed in the literature. However, due to limited validation of sampling and quenching procedures provided in previous publications, we thoroughly investigated the associated critical issues, such as (a) cellular integrity, (b) quenching efficiency, (c) cell separation at different centrifugation conditions and its influence on cell fitness, and (d) different washing procedures to prevent carryover of extracellular metabolites. Many metabolites of interest are also contained in the medium in large amounts, e.g. amino acids, making their intracellular quantification critical.

## Materials and methods

### Cell cultivation

Two CHO cell lines were used, T-CHO ATIII cells (GBF, Braunschweig, Germany) cultivated in serum-free CHO-S-SFM II medium (GIBCO, Invitrogen, Karlsruhe, Germany) and CHO K1 cells (University of Bielefeld, Germany) cultivated in amino acid rich TC-42 medium (TeutoCell, Bielefeld, Germany) in baffled shake flasks in a shaking incubator. Cell counting and determination of cell diameters were performed using an automated cell counter (Invitrogen, Darmstadt, Germany). Cell viability was verified using the trypan blue exclusion method. Cell recovery was defined as (total viable cell number after quenching)×100/(total viable cell number in initial sample).

### Determination of the energy charge

ATP, ADP, and AMP were analyzed in a luminometer (Promega, Mannheim, Germany) using the CellTiter-Glo, the ADP-Glo Kinase, and the AMP-Glo assays (Promega, Mannheim, Germany), respectively. For the ADP- and AMP-Glo assays, cells were lysed using the CelLytic M reagent (Sigma-Aldrich, Germany) before adding the assay reagents. The energy charge value was calculated as ([ATP] + ½×[ADP])/([ATP]+[ADP]+[AMP]).

### Evaluation of different washing procedures and carryover of media components

Carryover of extracellular metabolites from the culture medium was investigated without washing and after applying different washing procedures. Cell pellets were either resuspended in 50 ml quenching solution or rinsed once or twice with 50 ml quenching solution without re-suspension. After another centrifugation step and re-suspension in a small volume PBS, cell numbers were determined and extracellular metabolite amounts analyzed via HPLC as described previously [2] and related to the initial sample.

### Final protocol

(1) Precooling of 45 ml and 50 ml 0.9% saline quenching solution in an ice-water bath to 0°C for at least 1 hour. (2) Adding of 5 ml cell suspension to 45 ml 0.9% quenching solution and immediate mixing by inverting the tube. (3) Centrifugation at 2000 × g in a precooled centrifuge at 0°C for 1 min. (4) Careful decanting of the supernatant followed immediately by suction of residual liquid using a vacuum pump without touching the cell pellet. (5) Washing once by careful pouring of 50 ml precooled QS 50 ml on top of the cell pellet without resuspending the cells followed by repetition of steps (3) and (4). (6a) Immediate freezing by placing the tube in liquid nitrogen or (6b) determination of cell recovery.

## Results

Ice-cold 0.9% saline is a suitable quenching solution maintaining cellular integrity as reported previously [3]. However, longer incubation times at 0°C reduce cellular viability and should be avoided. The time from taking the sample (final protocol, step 2) to freezing the cell pellet in liquid nitrogen (final protocol, step 6a) is critical and should be kept to a minimum.

A rapid temperature shift and in addition a significant dilution of extracellular metabolites was achieved using a nine-fold excess of quenching solution. Efficient inactivation of metabolism was proven by a high and representative energy charge value of 0.82 (± 0.01, *n *= 3).

Separation of cells via centrifugation was incomplete due to required short centrifugal times. Thus, it is necessary to determine the cell recovery after quenching. However, from the average cell size estimation we conclude that centrifugation at short times provides a representative sample, although sampling was incomplete. Centrifugation time and speed, total volume and even the initial cell density in the cell suspension have an impact on the cell recovery after quenching. Centrifugation at 1000 × g and 2000 × g did not affect cell integrity. Higher centrifugal accelerations (3000 × g, 4000 × g) reduce cell viability. Above 2000 × g no further improvement in the cell recovery was obtained. Thus, centrifugation should be limited to 2000 × g to prevent unnecessary stress to the cells. Due to highly reproducible centrifugation, the cell recovery can be determined from a biological replicate (final protocol, step 6b).

Washing steps further reduce cell recovery. Rinsing the cell pellet affects cell recovery only little and much less than resuspending the cell pellet. Cell integrity was not impaired by different washing procedures. Reducing the carryover of metabolites contained in the medium is a prerequisite for their intracellular analysis. Using a nine-fold excess of quenching solution, contamination with medium components was very low (less than 0.3% of the initial metabolite amount was found for glucose, lactate, pyruvate, citrate, and all proteinogenic amino acids). Rinsing the cell pellet without re-suspending the cells further reduces the carryover of medium components efficiently. However, washing cannot completely prevent medium carryover. Washing by resuspending does not remove more metabolites than rinsing and should be avoided due to substantially reduced cell recovery.

## Conclusions

Ice-cold 0.9% saline was shown to be a suitable quenching solution maintaining cellular integrity. A rapid temperature shift was achieved using a nine-fold excess of quenching solution resulting in efficient inactivation of metabolism. The applied conditions result in a very low level of medium contamination. Rinsing the cell pellet without re-suspending the cells reduced medium carryover effectively. Separation of cells via centrifugation was incomplete due to required short centrifugal times. Thus, it is necessary to determine the cell recovery after quenching.
